# Supplementary material for: Early Selection of the Amino Acid Alphabet Was Adaptively Shaped by Biophysical Constraints of Foldability
Source: J Am Chem Soc. 2023 Feb 24;145(9):5320–9. doi: 10.1021/jacs.2c12987 (PMC10017022; doi:10.1021/jacs.2c12987)
Supplement: Supplementary file 1 — ja2c12987_si_001.pdf [file ja2c12987_si_001.pdf]

Supplementary Information

Early selection of the amino acid alphabet was adaptively shaped by biophysical constraints of foldability

Mikhail Makarov[1], Alma C. Sanchez Rocha[1], Robin Krystufek[2,3], Ivan Cherepashuk[1], Volha Dzmitruk[4], Tatsiana Charnavets[4], Anneliese M. Faustino,[5] Michal Lebl[3], Kosuke Fujishima[6,7], Stephen D. Fried*[5,8], and Klara Hlouchova*[1,3]

[1]Department of Cell Biology, Faculty of Science, Charles University, BIOCEV, Prague, 12843, Czech Republic

[2]Department of Physical Chemistry, Faculty of Science, Charles University, Prague, 12843, Czech Republic

[3]Institute of Organic Chemistry and Biochemistry, Czech Academy of Sciences, Prague, 16610, Czech Republic

[4]Institute of Biotechnology of the Czech Academy of Sciences, BIOCEV, 25250 Vestec, Czech Republic

[5]Department of Chemistry, Johns Hopkins University, Baltimore, Maryland 21212, United States

[6]Earth-Life Science Institute, Tokyo Institute of Technology, Tokyo, 1528550, Japan

[7]Graduate School of Media and Governance, Keio University, Fujisawa, 2520882, Japan

[8]Department of Biophysics, Johns Hopkins University, Baltimore, Maryland 21212, United States

* To whom correspondence may be addressed: klara.hlouchova@natur.cuni.cz, sdfried@jhu.edu

Table of contents

Figure S1. Molecular weight distribution of 25-mer combinatorial peptide libraries................1

Figure S2. Amino acid composition of 25-mer combinatorial peptide libraries estimated by HPLC amino acid analysis...2

Figure S3. The quantification of free amino acid groups in 10E and 11D peptide libraries using fluorescamine assay...3

Figure S4. Aggregation propensity of 25-mer combinatorial peptide libraries at different pH and low ionic strength..4

Figure S5. Aggregation propensity of 25-mer combinatorial peptide libraries at different pH and high ionic strength...5

Figure S6. The effect of metal ions on the secondary structure of 25-mer combinatorial peptide libraries...6

Table S1: Weights (mg) of protected amino acids in individual isokinetic mixtures used for synthesis 7

Supplementary Information

Table S2: Statistical analysis of relative solubilities at different pH and ionic strengths………8

Table S3: Statistical analysis of CD222/CD200 values at different pH values……….……12

Table S4: Statistical analysis of CD222/CD200 values at different 2,2,2-trifluoroethanol concentrations……………………………………………………………………………………..14

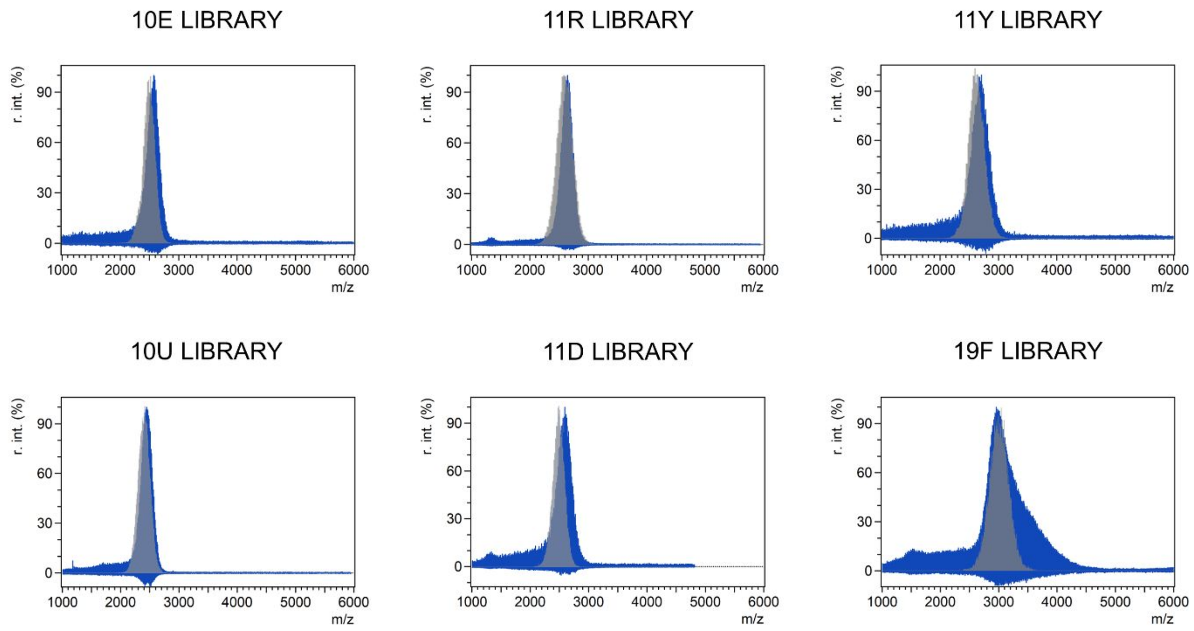

Supplementary Figure S1. Molecular weight distribution of 25-mer combinatorial peptide libraries estimated by MALDI-TOF-MS using 2,5-dihydrobenzoic acid (DHB) matrix. Observed m/z distribution is shown in blue, expected m/z distribution is shown as a gray overlay. Peptide libraries were dissolved in acetonitrile:water (1:1) mixture + 0.1 % (v/v) acetic acid. 19F peptide library was dissolved in acetonitrile:water (1:1) mixture.

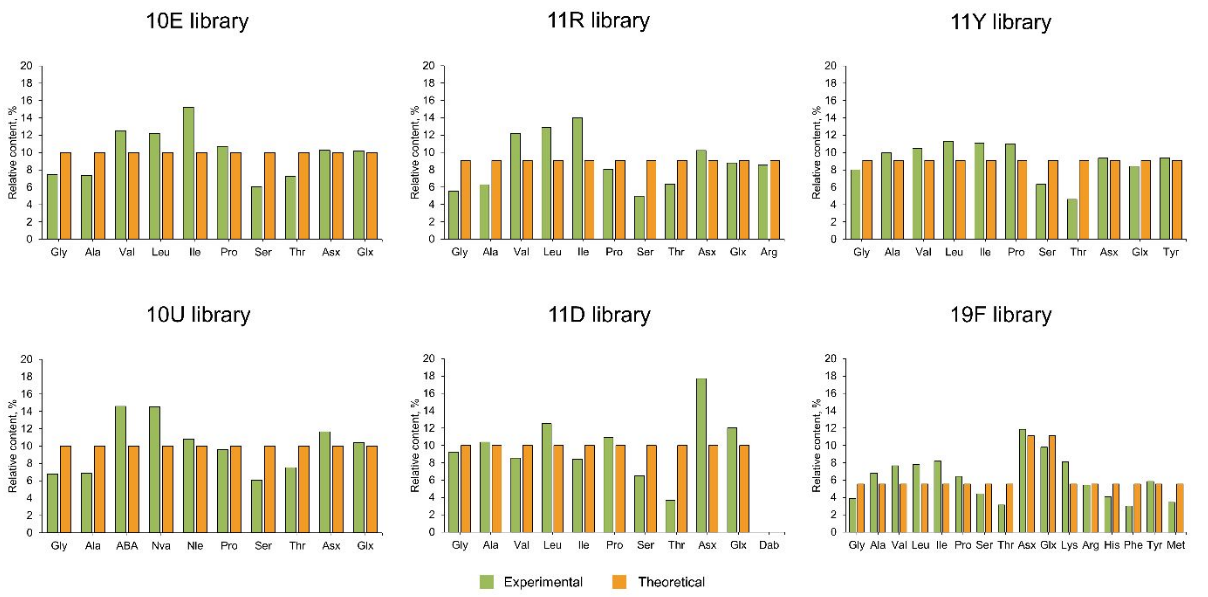

Supplementary Figure S2. Amino acid composition of 25-mer combinatorial peptide libraries determined by amino acid analysis.

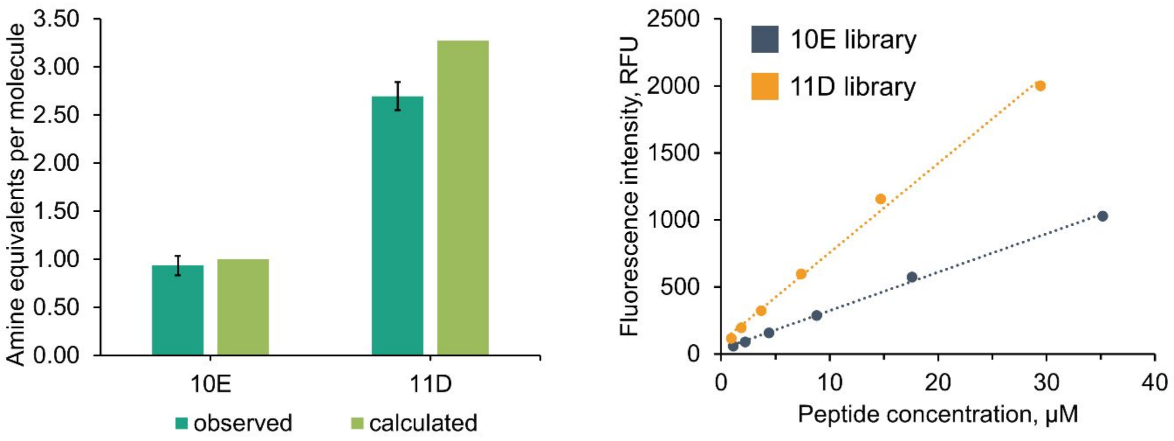

Supplementary Figure S3. The quantification of free amino acid groups in 10E and 11D peptide libraries using fluorescamine assay.

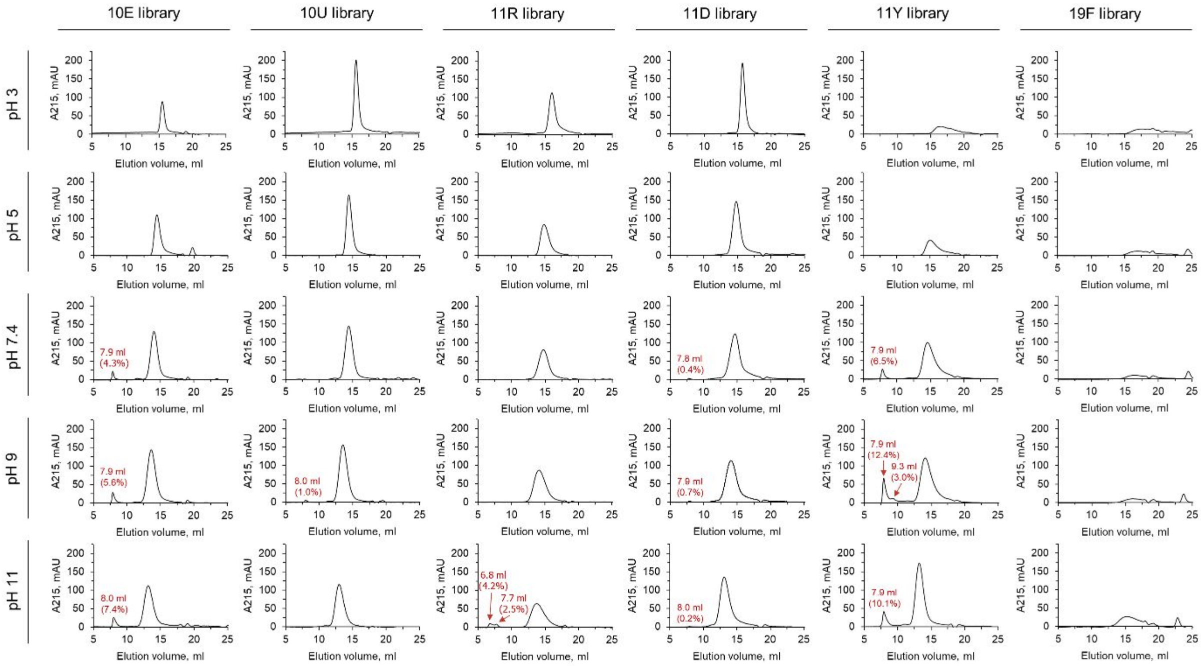

Supplementary Figure S4. Aggregation propensity of 25-mer combinatorial peptide libraries at different pH and low ionic strength (50 mM NaCl). Aggregation propensity was measured for 0.5 mg/ml nominal peptide library solutions in a series of 20 mM ABP buffers (pH 3–11) by size-exclusion chromatography.

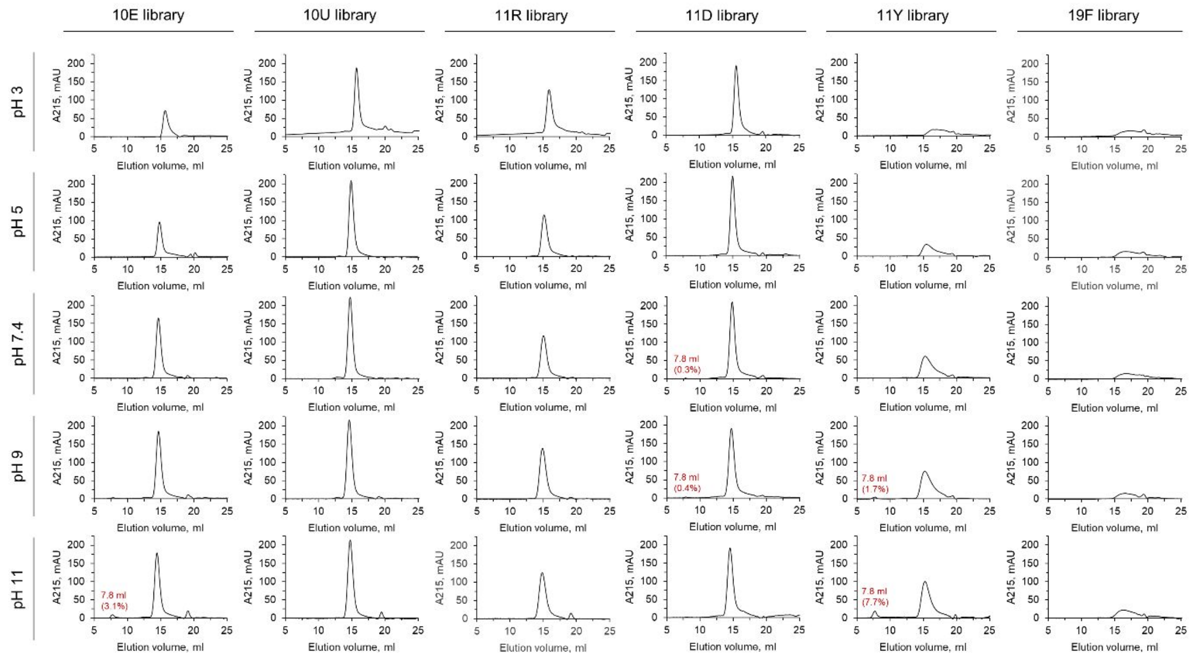

Supplementary Figure S5. Aggregation propensity of 25-mer combinatorial peptide libraries at different pH and high ionic strength (500 mM NaCl). Aggregation propensity was measured for 0.5 mg/ml nominal peptide library solutions in a series of 20 mM ABP buffers (pH 3–11) by size-exclusion chromatography.

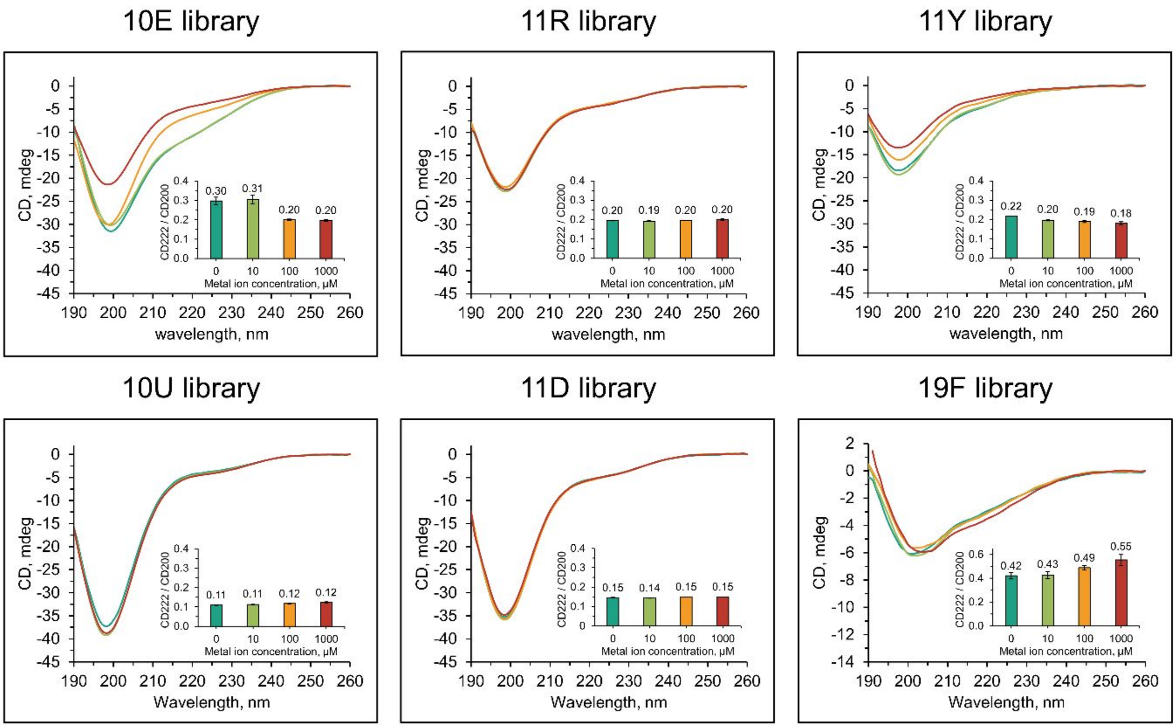

Supplementary Figure S6. The effect of metal ions on the secondary structure of 25-mer combinatorial peptide libraries. CD spectra were measured for 0.2 mg/ml nominal peptide library solutions in a series of 10 mM Tris buffers at pH 7.4 supplemented with 0 (green line); 10 (yellow line); 100 (orange line); and 1,000 (red line) μM mixture of NaCl, KCl, $MgCl_2$, $MnCl_2$, and $ZnCl_2$. The inlet graph shows the ratios of CD signal at 222 to 200 nm.

Supplementary Table S1: Weights (mg) and amounts (mmol) of protected amino acids in individual isokinetic mixtures used for synthesis. Mixtures were dissolved in a final volume listed in the last row yielding 0.3 M solution of amino acids in 0.3 M HOBt in DMF.

	19F		10E		11R		11Y		10U		11D	
	m mg	n mmol	m mg	n mmol	m mg	n mmol	m mg	n mmol	m mg	n mmol	m mg	n mmol
Fmoc-Ala	202	0.61	332	1.01	300	0.91	312	0.95	508	1.54	303	0.92
Fmoc-Asp(OtBu)	276	0.67	455	1.11	409	0.99	426	1.04	694	1.69	414	1.01
Fmoc-Glu(OtBu)	296	0.67	489	1.10	440	0.99	457	1.03	745	1.68	444	1.00
Fmoc-Phe	187	0.48										
Fmoc-Gly	164	0.55	270	0.91	243	0.82	253	0.85	412	1.39	246	0.83
Fmoc-His(Trt)	422	0.68										
Fmoc-Ile	1,171	3.31	1,934	5.47	1,740	4.92	1,809	5.12			1,759	4.98
Fmoc-Lys(Boc)	556	1.19										
Fmoc-Leu	335	0.95	553	1.56	497	1.41	517	1.46			503	1.42
Fmoc-Met	163	0.44										
Fmoc-Asn(Trt)	609	1.02										
Fmoc-Pro	278	0.78	460	1.29	414	1.16	430	1.21	702	1.98	418	1.18
Fmoc-Gln(Trt)	620	1.02										
Fmoc-Arg(Pmc)	807	1.22			1,199	1.81						
Fmoc-Ser(tBu)	203	0.53	336	0.88	302	0.79	314	0.82	512	1.34	306	0.80
Fmoc-Thr(tBu)	363	0.91	599	1.51	539	1.36	560	1.41	914	2.30	545	1.37
Fmoc-Val	731	2.15	1,208	3.56	1,086	3.20	1,130	3.33			1,099	3.24
Fmoc-Trp(Boc)	254	0.48										
Fmoc-Tyr(tBu)	363	0.79					560	1.22				
Fmoc-Nva									752	2.22		
Fmoc-Nle									775	2.19		
Fmoc-Aba									648	1.99		
Fmoc-Dab(Boc)											734	1.67
V / ml	61.5		61.3		61.2		61.4		39.7		55.8	

Supplementary table S2. Statistical analysis of relative solubilities at different pH and ionic strengths. The tables include ratios of relative solubilities and P-values. P-values are calculated using a two-tailed Student's t-test and assuming homoscedasticity.

(a) Statistical analysis of solubilities at low (50 mM NaCl) vs high (500 mM NaCl) ionic strengths

		10E	10U	11R	11D	11Y	19F
pH 3.0	Ratio	1.0264	1.0285	1.1484	1.0682	1.0560	1.4677
	P-value	0.3448	0.3946	0.0564	0.0214	0.4727	0.0172
pH 5.0	Ratio	0.9580	1.0137	0.9736	0.9656	0.9895	1.4433
	P-value	0.6268	0.6617	0.7776	0.0395	0.8594	0.0226
pH 7.4	Ratio	1.2833	0.9966	1.0013	0.9875	1.6230	1.0857
	P-value	0.0002	0.8585	0.9781	0.4112	0.0031	0.2538
pH 9.0	Ratio	0.9979	1.0239	1.1019	0.9945	1.4544	1.0753
	P-value	0.8370	0.1289	0.0263	0.4117	0.0160	0.5570
pH 11.0	Ratio	0.9881	0.9957	1.3588	1.0231	1.1160	0.9891
	P-value	0.4577	0.6999	2.1E-5	0.0546	0.4751	0.8085

(b) Statistical analysis of solubilities at low (50 mM NaCl) ionic strength at pH 3.0

		10E	10U	11R	11D	11Y	19F
10E	Ratio						
	P-value						
10U	Ratio	0.4673					
	P-value	2.1E-5					
11R	Ratio	0.5790	1.2391				
	P-value	4.0E-5	0.0028				
11D	Ratio	0.3778	0.8085	0.6524			
	P-value	3.3E-7	0.0007	3.1E-5			
11Y	Ratio	2.2921	4.9050	3.9584	6.0671		
	P-value	4.5E-6	4.7E-6	5.0E-6	1.5E-7		
19F	Ratio	0.9739	2.0842	1.6820	2.5780	0.4249	
	P-value	0.6288	0.0002	0.0007	2.0E-5	0.0008	

(c) Statistical analysis of solubilities at low (50 mM NaCl) ionic strength at pH 5.0

		10E	10U	11R	11D	11Y	19F
10E	Ratio						
	P-value						
10U	Ratio	0.4997					
	P-value	0.0001					
11R	Ratio	0.8370	1.6750				
	P-value	0.0958	0.0008				
11D	Ratio	0.4947	0.9901	0.5911			
	P-value	4.9E-5	0.5904	0.0004			
11Y	Ratio	2.0150	4.0324	2.4075	4.0729		
	P-value	0.0009	4.9E-6	0.0008	2.5E-7		
19F	Ratio	1.3055	2.6127	1.5598	2.6389	0.6479	
	P-value	0.0309	3.4E-5	0.0081	1.0E-5	0.0044	

(d) Statistical analysis of solubilities at low (50 mM NaCl) ionic strength at pH 7.4

		10E	10U	11R	11D	11Y	19F
10E	Ratio						
	P-value						
10U	Ratio	1.0024					
	P-value	0.9157					
11R	Ratio	1.4744	1.4709				
	P-value	0.0001	0.0001				
11D	Ratio	1.0098	1.0074	0.6849			
	P-value	0.6847	0.7210	0.0001			
11Y	Ratio	1.1801	1.1773	0.8004	1.1687		
	P-value	0.0025	0.0021	0.0052	0.0022		
19F	Ratio	4.1326	4.1229	2.8029	4.0927	3.5020	
	P-value	1.8E-6	1.1E-6	3.8E-5	1.0E-6	1.6E-5	

(e) Statistical analysis of solubilities at low (50 mM NaCl) ionic strength at pH 9.0

		10E	10U	11R	11D	11Y	19F
10E	Ratio						
	P-value						
10U	Ratio	0.9998					
	P-value	0.9490					
11R	Ratio	1.2767	1.2769				
	P-value	4.6E-5	2.3E-5				
11D	Ratio	1.0094	1.0096	0.7907			
	P-value	0.5400	0.3905	6.7E-6			
11Y	Ratio	0.9979	0.9982	0.7817	0.9886		
	P-value	0.6588	0.6219	0.0006	0.8537		
19F	Ratio	3.9955	3.9963	3.1296	3.9582	4.0037	
	P-value	9.5E-6	8.5E-6	3.1E-5	7.8E-6	2.0E-5	

(f) Statistical analysis of solubilities at low (50 mM NaCl) ionic strength at pH 11.0

		10E	10U	11R	11D	11Y	19F
10E	Ratio						
	P-value						
10U	Ratio	0.9840					
	P-value	0.3263					
11R	Ratio	0.9798	0.9957				
	P-value	0.0895	0.2452				
11D	Ratio	0.9798	0.9957	1.0000			
	P-value	0.1452	0.4924	0.2810			
11Y	Ratio	1.0313	1.0480	1.0526	1.0526		
	P-value	0.0860	0.0213	0.0070	0.0099		
19F	Ratio	3.4413	3.4971	3.5124	3.5124	3.3370	
	P-value	2.0E-6	8.9E-7	3.7E-7	4.5E-7	3.2E-6	

(g) Statistical analysis of solubilities at high (500 mM NaCl) ionic strength at pH 3.0

		10E	10U	11R	11D	11Y	19F
10E	Ratio						
	P-value						
10U	Ratio	0.4683					
	P-value	1.6E-5					
11R	Ratio	0.6478	1.3835				
	P-value	0.0024	0.0027				
11D	Ratio	0.3932	0.8397	0.6069			
	P-value	4.9E-6	0.0019	0.0003			
11Y	Ratio	2.3581	5.0360	3.6400	5.9976		
	P-value	7.3E-5	3.5E-6	0.0002	1.5E-6		
19F	Ratio	1.3926	2.9741	2.1497	3.5420	0.5906	
	P-value	0.0096	3.1E-5	0.0010	1.1E-5	0.0073	

(h) Statistical analysis of solubilities at high (500 mM NaCl) ionic strength at pH 5.0

		10E	10U	11R	11D	11Y	19F
10E	Ratio						
	P-value						
10U	Ratio	0.5287					
	P-value	0.0002					
11R	Ratio	0.8506	1.6087				
	P-value	0.1249	0.0010				
11D	Ratio	0.4986	0.9431	0.5862			
	P-value	9.6E-5	0.0519	0.0004			
11Y	Ratio	2.0812	3.9362	2.4468	4.1737		
	P-value	0.0012	4.8E-6	0.0007	6.7E-7		
19F	Ratio	1.9669	3.7201	2.3124	3.9446	0.9451	
	P-value	0.0031	2.1E-5	0.0015	7.9E-6	0.4946	

(i) Statistical analysis of solubilities at high (500 mM NaCl) ionic strength at pH 7.4

		10E	10U	11R	11D	11Y	19F
10E	Ratio						
	P-value						
10U	Ratio	0.7784					
	P-value	0.0003					
11R	Ratio	1.1505	1.4779				
	P-value	0.0209	0.0004				
11D	Ratio	0.7770	0.9982	0.6754			
	P-value	9.2E-5	0.8350	0.0003			
11Y	Ratio	1.4926	1.9174	1.2974	1.9208		
	P-value	0.0050	0.0006	0.0330	0.0005		
19F	Ratio	3.4963	4.4915	3.0391	4.4995	2.3425	
	P-value	1.6E-6	1.4E-6	5.7E-5	4.5E-7	0.0034	

(j) Statistical analysis of solubilities at high (500 mM NaCl) ionic strength at pH 9.0

		10E	10U	11R	11D	11Y	19F
10E	Ratio						
	P-value						
10U	Ratio	1.0257					
	P-value	0.0698					
11R	Ratio	1.4097	1.3743				
	P-value	0.0001	0.0003				
11D	Ratio	1.0059	0.9807	0.7136			
	P-value	0.6154	0.1297	0.0002			
11Y	Ratio	1.4544	1.4179	1.0318	1.4459		
	P-value	0.0125	0.0168	0.6691	0.0130		
19F	Ratio	4.3053	4.1973	3.0542	4.2800	2.9602	
	P-value	3.5E-7	1.1E-6	3.4E-5	5.1E-7	0.0041	

(k) Statistical analysis of solubilities at high (500 mM NaCl) ionic strength at pH 11.0

		10E	10U	11R	11D	11Y	19F
10E	Ratio						
	P-value						
10U	Ratio	0.9916					
	P-value	0.4210					
11R	Ratio	1.3474	1.3588				
	P-value	5.4E-5	4.1E-5				
11D	Ratio	1.0145	1.0231	0.7529			
	P-value	0.3492	0.1158	6.6E-5			
11Y	Ratio	1.1647	1.1746	0.8644	1.1481		
	P-value	0.2884	0.2646	0.4659	0.3213		
19F	Ratio	3.4451	3.4743	2.5568	3.3959	2.9578	
	P-value	1.4E-7	9.6E-8	3.3E-6	1.6E-7	0.0111	

Supplementary table S3. Statistical analysis of CD222/CD200 values at different pH values. The tables include ratios of CD222/CD200 values and P-values. P-values are calculated using a two-tailed Student's t-test and assuming homoscedasticity.

(a) Statistical analysis of CD222/CD200 values at pH 3.0

		10E	10U	11R	11D	11Y	19F
10E	Ratio						
	P-value						
10U	Ratio	1.4454					
	P-value	0.0102					
11R	Ratio	1.0564	0.7309				
	P-value	0.2795	0.0054				
11D	Ratio	1.3374	0.9253	1.2660			
	P-value	0.0159	0.0213	0.0102			
11Y	Ratio	0.9235	0.6389	0.8741	0.6905		
	P-value	0.2158	0.0076	0.0730	0.7528		
19F	Ratio	0.7829	0.5416	0.7410	0.5853	0.8477	
	P-value	0.0796	0.0170	0.0531	0.9173	0.1479	

(b) Statistical analysis of CD222/CD200 values at pH 5.0

		10E	10U	11R	11D	11Y	19F
10E	Ratio						
	P-value						
10U	Ratio	1.5078					
	P-value	0.0059					
11R	Ratio	1.0490	0.6957				
	P-value	0.1904	0.0012				
11D	Ratio	1.3382	0.8875	1.2757			
	P-value	0.0101	0.0249	0.0019			
11Y	Ratio	1.0940	0.7255	1.0429	0.8175		
	P-value	0.0928	0.0051	0.1186	0.5937		
19F	Ratio	0.5058	0.3354	0.4822	0.3379	0.4623	
	P-value	0.0760	0.0440	0.0695	0.6946	0.0651	

(c) Statistical analysis of CD222/CD200 values at pH 7.4

		10E	10U	11R	11D	11Y	19F
10E	Ratio						
	P-value						
10U	Ratio	3.0516					
	P-value	0.0004					
11R	Ratio	1.7835	0.5844				
	P-value	0.0017	0.0056				
11D	Ratio	2.4441	0.8009	1.3704			
	P-value	0.0003	0.0118	0.0101			
11Y	Ratio	1.2538	0.4109	0.7030	0.5130		
	P-value	0.0037	0.0006	0.0052	0.9884		
19F	Ratio	1.5215	0.4986	0.8531	0.6225	1.2135	
	P-value	0.0122	0.0130	0.1373	0.8600	0.0655	

(d) Statistical analysis of CD222/CD200 values at pH 9.0

		10E	10U	11R	11D	11Y	19F
10E	Ratio						
	P-value						
10U	Ratio	2.3852					
	P-value	0.0006					
11R	Ratio	0.9834	0.4123				
	P-value	0.2634	0.0002				
11D	Ratio	1.9297	0.8091	1.9623			
	P-value	0.0005	0.0096	3.0E-5			
11Y	Ratio	0.9257	0.3881	0.9413	0.4797		
	P-value	0.3894	0.0121	0.4754	0.9183		
19F	Ratio	0.9682	0.4059	0.9845	0.5017	1.0459	
	P-value	0.2465	0.0010	0.4470	0.9906	0.5910	

(e) Statistical analysis of CD222/CD200 values at pH 11.0

		10E	10U	11R	11D	11Y	19F
10E	Ratio						
	P-value						
10U	Ratio	2.2522					
	P-value	0.0003					
11R	Ratio	0.7160	0.3179				
	P-value	0.0240	0.0043				
11D	Ratio	1.9858	0.8817	2.7734			
	P-value	0.0002	0.0204	0.0049			
11Y	Ratio	0.9523	0.4228	1.3301	0.4796		
	P-value	0.0564	0.0005	0.0317	0.9635		
19F	Ratio	0.9157	0.4066	1.2790	0.4611	0.9616	
	P-value	0.0088	0.0002	0.0397	0.9408	0.0774	

Supplementary table S4. Statistical analysis of CD222/CD200 values at different 2,2,2-trifluoroethanol concentrations. The tables include ratios of CD222/CD200 values and P-values. P-values are calculated using a two-tailed Student's t-test and assuming homoscedasticity.

(a) Statistical analysis of CD222/CD200 values at 0% (v/v) 2,2,2-trifluoroethanol

		10E	10U	11R	11D	11Y	19F
10E	Ratio						
	P-value						
10U	Ratio	3.0516					
	P-value	0.0004					
11R	Ratio	1.7910	0.5869				
	P-value	0.0013	0.0045				
11D	Ratio	2.4441	0.8009	1.3647			
	P-value	0.0003	0.0118	0.0075			
11Y	Ratio	1.2538	0.4109	0.7000	0.5130		
	P-value	0.0037	0.0006	0.0039	0.9884		
19F	Ratio	1.6823	0.5513	0.9393	0.6883	1.3417	
	P-value	0.0560	0.1169	0.7543	0.8254	0.1796	

(b) Statistical analysis of CD222/CD200 values at 10% (v/v) 2,2,2-trifluoroethanol

		10E	10U	11R	11D	11Y	19F
10E	Ratio						
	P-value						
10U	Ratio	3.3216					
	P-value	0.0005					
11R	Ratio	2.0610	0.6205				
	P-value	0.0030	0.0154				
11D	Ratio	2.8413	0.8554	1.3786			
	P-value	0.0006	0.0032	0.0292			
11Y	Ratio	1.2974	0.3906	0.6295	0.4566		
	P-value	0.0090	0.0010	0.0092	0.9237		
19F	Ratio	1.6901	0.5088	0.8201	0.5948	1.3027	
	P-value	0.0027	0.0023	0.0588	0.8806	0.0128	

(c) Statistical analysis of CD222/CD200 values at 20% (v/v) 2,2,2-trifluoroethanol

		10E	10U	11R	11D	11Y	19F
10E	Ratio						
	P-value						
10U	Ratio	2.6675					
	P-value	0.0001					
11R	Ratio	1.6638	0.6237				
	P-value	0.0006	0.0025				
11D	Ratio	2.3442	0.8788	1.4089			
	P-value	0.0002	0.0341	0.0047			
11Y	Ratio	0.8460	0.3171	0.5085	0.3609		
	P-value	0.0032	0.0002	0.0006	0.7995		
19F	Ratio	0.6663	0.2498	0.4005	0.2843	0.7876	
	P-value	0.0005	0.0001	0.0003	0.7014	0.0023	

(d) Statistical analysis of CD222/CD200 values at 30% (v/v) 2,2,2-trifluoroethanol

		10E	10U	11R	11D	11Y	19F
10E	Ratio						
	P-value						
10U	Ratio	2.2870					
	P-value	4.9E-5					
11R	Ratio	1.2366	0.5407				
	P-value	0.0074	0.0021				
11D	Ratio	1.9516	0.8534	1.5782			
	P-value	0.0003	0.0138	0.0038			
11Y	Ratio	0.6514	0.2849	0.5268	0.3338		
	P-value	0.0007	0.0002	0.0009	0.7630		
19F	Ratio	0.2595	0.1134	0.2098	0.1330	0.3983	
	P-value	0.0097	0.0068	0.0085	0.4754	0.0146	

(e) Statistical analysis of CD222/CD200 values at 40% (v/v) 2,2,2-trifluoroethanol

		10E	10U	11R	11D	11Y	19F
10E	Ratio						
	P-value						
10U	Ratio	2.1465					
	P-value	0.0015					
11R	Ratio	1.0300	0.4799				
	P-value	0.5512	0.0055				
11D	Ratio	1.8074	0.8420	1.7547			
	P-value	0.0019	0.0123	0.0076			
11Y	Ratio	0.5943	0.2769	0.5770	0.3288		
	P-value	0.0032	0.0008	0.0048	0.7472		
19F	Ratio	0.1359	0.0633	0.1320	0.0752	0.2287	
	P-value	0.0184	0.0157	0.0182	0.3865	0.0229	

(f) Statistical analysis of CD222/CD200 values at 50% (v/v) 2,2,2-trifluoroethanol

		10E	10U	11R	11D	11Y	19F
10E	Ratio						
	P-value						
10U	Ratio	2.0240					
	P-value	0.0010					
11R	Ratio	0.9415	0.4652				
	P-value	0.2231	0.0036				
11D	Ratio	1.6898	0.8349	1.7948			
	P-value	0.0014	0.0136	0.0052			
11Y	Ratio	0.5706	0.2819	0.6060	0.3377		
	P-value	0.0007	0.0002	0.0027	0.7691		
19F	Ratio	0.1174	0.0580	0.1246	0.0694	0.2057	
	P-value	0.0008	0.0007	0.0008	0.4596	0.0010	

(g) Statistical analysis of CD222/CD200 values at 60% (v/v) 2,2,2-trifluoroethanol

		10E	10U	11R	11D	11Y	19F
10E	Ratio						
	P-value						
10U	Ratio	1.9533					
	P-value	0.0010					
11R	Ratio	0.9008	0.4612				
	P-value	0.0515	0.0016				
11D	Ratio	1.6126	0.8256	1.7901			
	P-value	0.0019	0.0146	0.0026			
11Y	Ratio	0.5535	0.2834	0.6145	0.3433		
	P-value	0.0003	4.2E-5	0.0011	0.7827		
19F	Ratio	0.1113	0.0570	0.1236	0.0690	0.2011	
	P-value	8.0E-6	5.7E-6	1.4E-5	0.4779	6.7E-6	

(h) Statistical analysis of CD222/CD200 values at 70% (v/v) 2,2,2-trifluoroethanol

		10E	10U	11R	11D	11Y	19F
10E	Ratio						
	P-value						
10U	Ratio	1.8746					
	P-value	0.0025					
11R	Ratio	0.8534	0.4553				
	P-value	0.0475	0.0029				
11D	Ratio	1.5377	0.8203	1.8019			
	P-value	0.0037	0.0106	0.0041			
11Y	Ratio	0.5533	0.2952	0.6483	0.3598		
	P-value	0.0007	0.0001	0.0028	0.8018		
19F	Ratio	0.0961	0.0513	0.1126	0.0625	0.1736	
	P-value	0.0049	0.0044	0.0051	0.4219	0.0058	

(i) Statistical analysis of CD222/CD200 values at 80% (v/v) 2,2,2-trifluoroethanol

		10E	10U	11R	11D	11Y	19F
10E	Ratio						
	P-value						
10U	Ratio	1.7646					
	P-value	0.0070					
11R	Ratio	0.8485	0.4809				
	P-value	0.0632	0.0039				
11D	Ratio	1.4690	0.8325	1.7312			
	P-value	0.0104	0.0245	0.0049			
11Y	Ratio	0.5381	0.3049	0.6341	0.3663		
	P-value	0.0014	0.0002	0.0026	0.8132		
19F	Ratio	0.0734	0.0416	0.0864	0.0499	0.1363	
	P-value	0.0011	0.0010	0.0011	0.4384	0.0012	

(j) Statistical analysis of CD222/CD200 values at 90% (v/v) 2,2,2-trifluoroethanol

		10E	10U	11R	11D	11Y	19F
10E	Ratio						
	P-value						
10U	Ratio	1.6794					
	P-value	0.0091					
11R	Ratio	0.8393	0.4997				
	P-value	0.0550	0.0029				
11D	Ratio	1.3614	0.8106	1.6221			
	P-value	0.0213	0.0201	0.0052			
11Y	Ratio	0.5214	0.3105	0.6213	0.3830		
	P-value	0.0143	0.0063	0.0217	0.7891		
19F	Ratio	0.0184	0.0110	0.0220	0.0135	0.0353	
	P-value	0.0270	0.0266	0.0272	0.3064	0.0279	
